# The Great “Salve” Certificate

**Published:** 1861-01

**Authors:** Sawny Green’un


**THE GREAT "SALVE" CERTIFICATE.**—

DEAR DOCTOR: I will be one hundred and seventy-five years old next October. For ninety-four years I have been an invalid, unable to move except when stirred with a lever. But a year age last Thursday, I heard of the Granicular Syrup. I bought a bottle, smelt of the cork, and found myself a new man. I can now run twelve and a half miles an hour, and throw nineteen double somersets without stopping.

P. S.—A little of your Alicumstoutum Salve applied to a wooden leg, reduced a compound fracture in nineteen minutes, and is now covering the limb with a fresh cuticle of white gum pine bark. SAWNY GREEN'UN.

